# Novel Potential Application of Chitosan Oligosaccharide for Attenuation of Renal Cyst Growth in the Treatment of Polycystic Kidney Disease

**DOI:** 10.3390/molecules25235589

**Published:** 2020-11-27

**Authors:** Nutthapoom Pathomthongtaweechai, Sunhapas Soodvilai, Rath Pichyangkura, Chatchai Muanprasat

**Affiliations:** 1 Chakri Naruebodindra Medical Institute, Faculty of Medicine Ramathibodi Hospital, Mahidol University, Bang Phli, Samut Prakan 10540, Thailand; nutthapoom.pat@mahidol.edu; 2 Department of Physiology, Faculty of Science, Mahidol University, Ratchathewi, Bangkok 10400, Thailand; sunhapas.soo@mahidol.ac.th; 3 Department of Biochemistry, Faculty of Science, Chulalongkorn University, Pathumwan, Bangkok 10330, Thailand; prath@chula.ac.th

**Keywords:** chitosan oligosaccharide, renal cyst, polycystic kidney disease, AMP-activated protein kinase, drug discovery

## Abstract

Chitosan oligosaccharide (COS), a natural polymer derived from chitosan, exerts several biological activities including anti-inflammation, anti-tumor, anti-metabolic syndrome, and drug delivery enhancer. Since COS is vastly distributed to kidney and eliminated in urine, it may have a potential advantage as the therapeutics of kidney diseases. Polycystic kidney disease (PKD) is a common genetic disorder characterized by multiple fluid-filled cysts, replacing normal renal parenchyma and leading to impaired renal function and end-stage renal disease (ESRD). The effective treatment for PKD still needs to be further elucidated. Interestingly, AMP-activated protein kinase (AMPK) has been proposed as a drug target for PKD. This study aimed to investigate the effect of COS on renal cyst enlargement and its underlying mechanisms. We found that COS at the concentrations of 50 and 100 µg/mL decreased renal cyst growth without cytotoxicity, as measured by MTT assay. Immunoblotting analysis showed that COS at 100 µg/mL activated AMPK, and this effect was abolished by STO-609, a calcium/calmodulin-dependent protein kinase kinase beta (CaMKKβ) inhibitor. Moreover, COS elevated the level of intracellular calcium. These results suggest that COS inhibits cyst progression by activation of AMPK via CaMKKβ. Therefore, COS may hold the potential for pharmaceutical application in PKD.

## 1. Introduction

Polycystic kidney disease (PKD) is a common genetic disorder with a predicted prevalence of over 10 million people worldwide, according to several clinical studies in the USA, Europe, and Japan. Approximately one out of 1000 live births are affected by PKD [1,2,3]. In the autosomal dominant trait form, which is the more prevalent form of PKD, it is caused by the mutation in either one of two genes, *PKD1* and *PKD2*, which encode for polycystin 1 (PC1) and polycystin 2 (PC2), respectively [4]. Lack of either PC1 or PC2 diminishes flow-sensitive calcium sensing and disrupts the homeostasis of intracellular calcium and cyclic adenosine monophosphate (cAMP) signaling in renal tubular cells [5,6]. PKD is characterized by multiple renal fluid-filled cysts that replace normal renal parenchyma bilaterally, along the renal tubules [7,8]. The progression of cyst enlargement causes distortion of the renal architecture and impairs renal function, eventually leading to end-stage renal failure (ESRD) in a majority of PKD patients [2,7,8,9]. In PKD, the decreased levels of intracellular calcium and the increased levels of intracellular cAMP lead to two pathophysiological events: (i) cyclic adenosine monophosphate (cAMP)-induced chloride secretion, in part driven by cystic fibrosis transmembrane conductance regulator (CFTR), and (ii) increased cell proliferation, at least in part mediated by mammalian target of rapamycin complex 1 (mTORC1) [10,11,12,13,14].

Of particular significance, both CFTR and mTORC1 are modulated by AMP-activated protein kinase (AMPK), a heterotrimeric protein complex that functions as a highly conserved cellular energy sensor and plays a pivotal role in the regulation of a variety of metabolic pathways. AMPK controls the energy balance by suppressing the anabolic pathway to decrease ATP consumption, such as inhibition of lipogenesis, gluconeogenesis, and cell growth, and promoting catabolism to increase ATP production, such as stimulation of fatty acid oxidation, glycolysis, and autophagy [15,16,17]. AMPK is ubiquitously expressed in the kidney to orchestrate the regulation of renal physiological and pathophysiological processes, including ion transport, energy metabolism, and disease conditions (e.g., diabetes, renal injury, renal fibrosis, lupus nephritis, and PKD) [18,19]. In PKD patients and animal models, the reduction of the level of AMPK and defective aerobic glycolysis, which is a key feature of proliferative tissues, have been shown [20]. However, the enhanced AMPK activation by 2-deoxy-d-glucose (2-DG) could reverse the abnormal glucose metabolism and cystic phenotype in PKD mice [20]. In addition, metformin, a known AMPK activator and type 2 diabetic drug, has been revealed to slow renal cyst growth in Madin–Darby canine kidney (MDCK) cells via the activation of AMPK [21]. Therefore, the compounds acting as AMPK activators are considered as promising potential drug candidates for PKD.

Chitosan oligosaccharide (COS) is a degraded oligomer of chitosan, a biocompatible linear polymer of β-(1→4)-linked D-glucosamine (GlcN, deacetylated unit D), which is prepared by the deacetylation of chitin, the second-most plentiful natural polymer in the world and abundantly found in the shell of crustaceans (e.g., crabs, shrimps, and lobsters) [22,23]. Its water soluble, thermally stable, non-toxic, non-allergenic, biodegradable, and chemically modifiable properties have brought numerous benefits to its pharmaceutical applications as nutraceuticals or food supplements [24,25]. COS exerts innumerable biological effects including anti-inflammation, anti-cancer, anti-metabolic syndrome, and anti-microbial [22,23]. Moreover, it can serve as a vehicle for drug delivery by modification with nanoparticles [22,25,26,27]. After degradation, the kidney is one of the major sites for tissue distribution and elimination of COS, as demonstrated in a rat model in which approximately 80% of COS was excreted in urine within 11–15 days [28,29]. It has also been reported that COS has a renoprotective effect in drug-induced nephrotoxicity and renal impairment, implicating the pivotal roles of COS in renal diseases [30,31]. Our research group has recently revealed that COS stimulated the activation of AMPK and suppressed intestinal inflammation and mucosal damage in intestinal epithelial cells (IECs) and mouse models of inflammatory bowel disease (IBD) [32]. However, the effect of COS on renal cyst progression has never been elucidated.

Herein, it is therefore hypothesized that COS may enhance the activation of AMPK and inhibit renal cyst enlargement. The aim of this study is to evaluate the effects of COS on cyst development in an in vitro cyst growth model of PKD using an MDCK cyst model due to its capability to initiate cyst formation and secrete chloride secretion in response to cAMP stimulation. Furthermore, the mechanisms by which COS attenuate cyst progression were investigated.

## 2. Results

### 2.1. Effect of COS on Renal Cyst Progression

**Figure 1:**
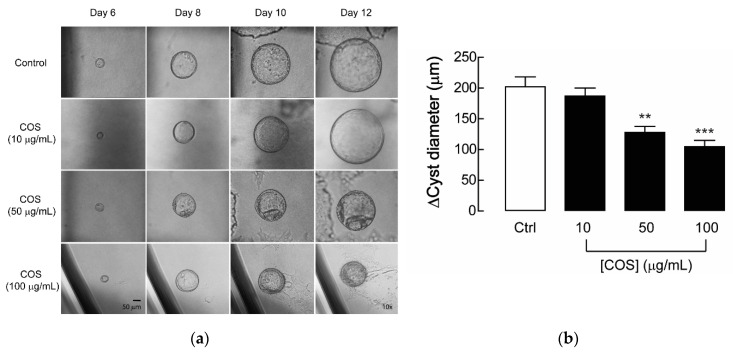
Effect of chitosan oligosaccharide (COS) on cyst progression in Madin–Darby canine kidney (MDCK) cyst model. (**a**) Representative micrographs show the MDCK cysts in 3D collagen gel bathed with culture medium containing forskolin with COS at the indicated concentrations or 1% acetic acid (vehicle control) at the indicated days after seeding. Scale bar = 50 μm; magnification = ×10. (**b**) Bar graphs represent the change of diameters of MDCK cysts by subtracting the outer diameters at day 12 with those at day 6 after seeding, in the presence of COS at indicated concentrations or 1% acetic acid (vehicle control) in forskolin-induced MDCK cyst model. Data are expressed as mean ± S.E.M. (*n* = 36–49 cysts per condition). ** *p* < 0.01; *** *p* < 0.001 compared with control.

In this study, a 3-dimensional (3D) MDCK cyst model was used for investigating the effect of COS on renal cyst enlargement in response to forskolin, an adenylate cyclase activator that enhances the level of intracellular cAMP to form renal cysts suspended in collagen. After cyst initiation, COS (MW ~5000 Da: degree of deacetylation of 90%) dissolved in 1% acetic acid at the concentrations of 10, 50, or 100 µg/mL were added into the culture medium from day 6 to day 12 in the forskolin-induced MDCK cyst model. The results revealed that the cysts treated with COS at concentrations of 50 and 100 µg/mL were significantly smaller in size when compared with the control group. The 1% acetic acid was represented as a negative control, which had no effect on MDCK cyst growth, while CFTR_inh_-172, a CFTR inhibitor, was used as a positive control and slowed the cyst growth rate to ~15% (data not shown), reflecting the reduction of MDCK cyst growth in these experimental groups. Meanwhile, COS at 10 µg/mL had no effect (Figure 1). These results indicate that COS treatment attenuates progression of renal cyst growth in the 3D MDCK cyst model.

### 2.2. Non-Cytotoxic Effect of COS in MDCK Cells

**Figure 2:**
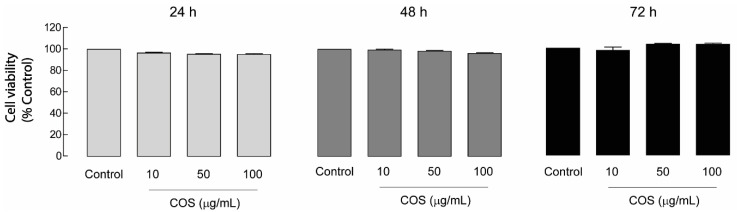
Effect of COS on MDCK cell viability. MDCK cells were seeded onto 96-well plates and incubated with COS at 0, 10, 50, or 100 µg/mL for 24, 48, or 72 h. MTT assays were performed to evaluate the effect of COS on MDCK cell viability. Data are expressed as mean of percent cell viability ± S.E.M. (*n* = 3–4).

Prior to the subsequent experiments, it was necessary to rule out whether the inhibitory effect of COS on cyst progression resulted from COS-induced cytotoxicity. In addition, cell toxicity is a crucial concern for developing nutraceuticals. Therefore, the effects of COS on cell viability were assessed. In this study, MDCK cell viability was evaluated in the presence of COS at various concentrations (10, 50, and 100 µg/mL) by using an MTT assay at 24, 48, or 72 h. The result showed that COS at all concentrations did not affect MDCK cell viability at all time courses (Figure 2). These results suggest that the inhibitory effect of COS on MDCK cyst growth may be not due to the cytotoxic effect of COS.

### 2.3. AMPK Activation by COS in MDCK Cells

**Figure 3:**
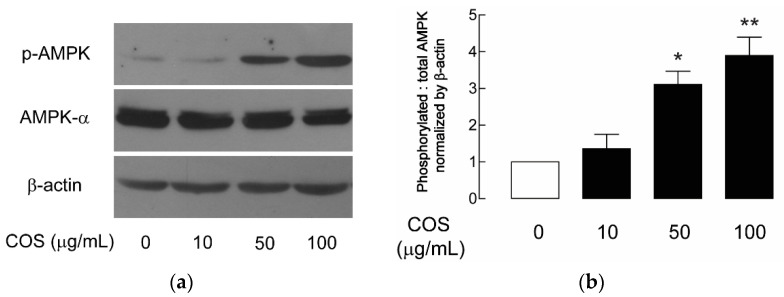
Effect of COS on AMPK activation in MDCK cells. (**a**) Representative immunoblots of five separate experiments for the expression of p-AMPK, AMPK-α, and β-actin were performed. MDCK cells were incubated with COS at 10, 50, or 100 µg/mL or vehicle for 24 h before collecting samples for immunoblotting analysis. (**b**) Densitometry analysis of p-AMPK corrected by total AMPK and β-actin is depicted. Data are expressed as mean ± S.E.M. (*n* = 5). * *p* < 0.05; ** *p* < 0.01 compared with control.

The activation of AMPK is known to inhibit CFTR-mediated chloride secretion and mTOR-mediated cell proliferation, which are two underlying mechanisms of renal cyst growth [11,12,13,21]. Previously, the AMPK activators, including metformin and pranlukast, were shown to slow MDCK cyst progression [21,33]. Therefore, the effect of COS on AMPK activation was investigated using immunoblotting. The α1-subnit of AMPK is the abundant catalytic isoform in the kidney. Phosphorylation at threonine (Thr)-172 within the activation loop or T-loop, the region in kinase domain of α subunit, is required for the activation of AMPK [15,16]. Immunoblotting using antibodies specific for phosphorylated AMPK (p-AMPK) was performed to investigate the effect of COS on AMPK activity. After treatment with COS for 24 h, it was revealed that COS enhanced the activation of AMPK in a concentration-dependent manner with the maximal effect being observed at 100 µg/mL, implicating the role of COS as an AMPK activator in MDCK cells (Figure 3).

### 2.4. Upstream Target of COS-Induced AMPK Activation

**Figure 4:**
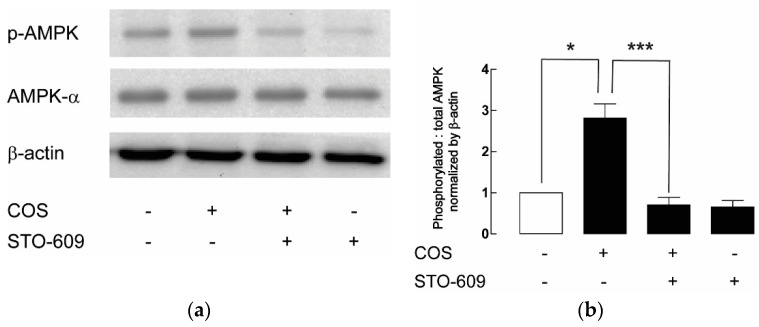
Evaluation of CaMKKβ as an upstream of COS-induced AMPK activation. (**a**) Representative immunoblots of five separate experiments were depicted. Samples were collected after 24-h treatment with 100 µg/mL of COS with or without 5 µM of STO-609. The symbol “+” represents the presence of the compounds (COS or STO-609), while “-“ exemplifies the absence of the compounds. (**b**) Densitometric analysis of p-AMPK corrected by total AMPK and β-actin is depicted. Data are expressed as mean ± S.E.M. (*n* = 5). * *p* < 0.05, *** *p* < 0.001.

**Figure 5:**
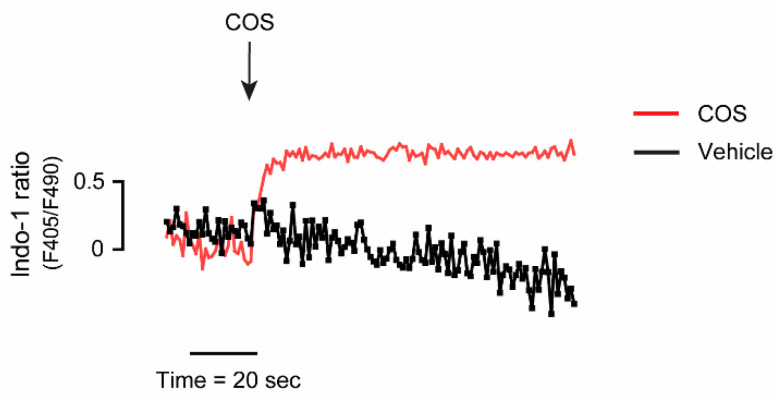
Effect of COS on the level of intracellular calcium. Representative tracing of indo-1 fluorescence ratio (emitted at 405 nm and 490 nm; F405/F490) was shown. After indo-1 loading, MDCK cells were suspended in buffers supplemented with Ca^2+^ (1 mM). During continual measurement of indo-1 fluorescence, COS (100 µg/mL) or 1% acetic acid (vehicle control) was added into the solutions.

The phosphorylation of Thr-172 of AMPK is mediated by two upstream kinases, including (i) calcium/calmodulin-dependent protein kinase kinase beta (CaMKKβ), stimulated by an increase in the level of intracellular calcium, and (ii) liver kinase B1 (LKB1), in response to an increase in the ratio of adenosine monophosphate (AMP) to adenosine triphosphate (ATP) [15,16]. In this study, we investigated whether COS-induced AMPK activation was mediated through CaMKKβ. Immunoblottings of p-AMPK after 24-h treatment with COS with or without cotreatment with 5 µM of STO-609, an inhibitor of CaMKKβ, were performed. In the presence of COS and STO-609, the phosphorylation of AMPK was decreased when compared with that of COS alone. In other words, STO-609 could suppress the stimulatory effect of COS on AMPK activation, suggesting that COS induces AMPK activation through CaMKKβ in MDCK cells (Figure 4). As CaMKKβ-mediated AMPK activation is triggered by an increase in intracellular calcium, the effect of COS on the level of intracellular calcium was evaluated. The indo-1 fluorescence ratio, which is the ratio of emitted fluorescence at 405 nm (calcium-bound indo-1) to 490 nm (calcium-free indo-1), was used as an indicator of intracellular calcium level. It was found that COS at 100 µg/mL stimulated an increase of intracellular calcium in MDCK cells compared with the vehicle control (Figure 5). Taken together, our results suggest that COS inhibits renal cyst growth through the activation of AMPK and the elevation of intracellular calcium.

## 3. Discussion

The strategies hitting two pathological processes, including massive chloride secretion and tremendously uncontrolled cell proliferation, have gained much attention for the therapeutic approaches in PKD. Several candidates have inhibitory effects on either of these two phenomena, such as small-molecule CFTR inhibitors (i.e., thiazolidinone, glycine hydrazide, tetrazolo-CFTR_inh_-172, and Ph-GlyH-101), natural extracts and their derivatives (i.e., stevioside, steviol, curcumin), and repositioning drugs (i.e., pranlukast and metformin) [21,33,34,35,36,37]. Particularly, metformin was raised to be a renal cyst inhibitor and an AMPK activator, which results in the inhibition of transepithelial fluid secretion and renal epithelial cell proliferation, implicating that the AMPK activator may serve as a drug for renal cyst inhibition [21]. Formerly, our research group has revealed that pranlukast, an anti-asthmatic drug, exerts an inhibitory effect on renal cyst development through the activation of AMPK, suggesting the potential application of pranlukast for PKD therapy [33].

Compared with chitosan, COS has improved physical and chemical properties, including water solubility, low viscosity, non-toxicity, biocompatibility, and biodegradability [22,24,25]. Therefore, COS has recently been paid increasing attention from a wide range of research interests, particularly in the field of drugs and foods as food additives, owing to its biological and pharmacological activities such as anti-inflammatory, anti-oxidant, anti-bacterial, anti-tumor, and immune-modulating effects [22,24,25]. In view of this study, we demonstrated that COS with the average MW of 5000 Da, which had more AMPK-enhancing effect than those with higher MW in our previous work in IECs, and DD of ~90% ameliorated forskolin-induced cyst swelling in MDCK cyst model via the activation of AMPK, proposing for the first time that COS is a novel class of the AMPK activator in MDCK cells and a potential renal cyst growth inhibitor. The involvement of AMPK and COS-induced inhibition of cyst growth may be addressed by using the AMPK inhibitor compound C or dorsomorphin. In the MDCK cyst model, compound C should be added to investigate whether COS-induced inhibition of cyst growth could be reversed by compound C. However, the limitation of compound C is its specificity to AMPK, as it also inhibits several kinases other than AMPK [38]. As several lines of evidence have capitulated for the anti-inflammatory effect of COS and the suppression of inflammatory processes by AMPK, COS might exert the inhibitory effect on MDCK cyst progression by AMPK-mediated anti-inflammatory activities [22,32,39,40]. Previously, we have reported that COS stimulates the activation of AMPK and inhibits nuclear factor kappa-light-chain-enhancer of activated B cells (NF-κB) in intestinal epithelial cells [32]. In addition, COS has been shown to attenuate tumor necrosis factor alpha (TNF-α)-induced inducible nitric oxide synthase (iNOS) and cyclooxygenase 2 (COX-2) expressions in synoviocytes [32,39]. It is possible that the inhibitory effects of COS via AMPK activation might be relevant to NF-κB or other downstream proteins of TNF-α. However, in our setting, we used forskolin, which mediates the AC/cAMP/protein kinase A (PKA)/cAMP response element-binding protein (CREB) signaling cascade, separately from the NF-κB-induced inflammatory pathway.

Since PKD is caused by PC1 and/or PC2 defects, which result in decreases in the levels of intracellular calcium, another therapeutic approach of PKD is to increase intracellular calcium [5,6]. In this study, we demonstrated that COS elevated the level of intracellular calcium in MDCK cells, and COS-induced AMPK activation was dependent on CaMKKβ activity. However, the role of LKB1 in AMPK activation was not further addressed, because STO-609 entirely abolished COS-induced AMPK activation. Our study demonstrated that COS inhibited renal cyst progression by increasing the intracellular calcium followed by CaMKKβ activation and AMPK activation. For the detailed mechanism, our group has previously suggested that COS-provoked AMPK activation in human colonic adenocarcinoma T84 cells is associated with calcium release from the endoplasmic reticulum (ER) and mitochondria through the calcium-sensing receptor (CaSR)-phospholipase C (PLC)-inositol triphosphate (IP3) receptor channel pathway, as well as with the assembly of epithelial tight junctions mediated by extracellular calcium [32]. CaSR, a G protein-coupled receptor (GPCR) playing a role in calcium homeostasis, is differentially expressed in particular segments of the nephron [41,42]. Indeed, it is found at the basolateral side of the thick ascending limb and distal convoluted tubules, as well as at the apical side of the proximal tubule and inner medullary collecting duct [42,43,44,45]. It is also expressed in MDCK cells and serves as the regulator of tight junction assembly [46,47,48]. In PKD, the activation of CaSR results in the reduction of cAMP levels and subsequent inhibition of renal cyst growth [41]. Calcimimetics, the allosteric modulators of CaSR, slow late-stage cyst progression in ADPKD rat by enhancing the level of intracellular calcium [41]. It is possible that COS-induced AMPK activation in MDCK cells may share a similar mechanism with T84 cells. As COS is distributed and eliminated in the kidney, and it also exerts a renoprotective effect in drug-induced renal injury, which is consistent with our study that COS at indicated concentrations showed no toxicity in MDCK cells, COS may exploit the therapeutic properties for use in renal diseases. Interestingly, COS can facilitate drug or nucleotide delivery systems with the aim of the improvement of drug bioavailability and lessened adverse effects [26,27,49,50]. COS and chitosan derivatives may serve as delivery carriers as nanoparticles or micelles [26,27,39,40,51]. Zidovudine (AZT), an anti-retroviral drug, is used for slowing the progression of human immunodeficiency virus-associated nephropathy (HIVAN) to ESRD in HIV-infected patients [52]. However, after administration, AZT has a very short half-life and is rapidly eliminated in human circulation and the kidney [52]. To surpass these limits, the conjugation of AZT with COS prolongs the mean retention time and has sustained its release, so that AZT–COS can accumulate in the mouse kidney, suggesting the potential of COS in a renal-targeting drug delivery system [50,52]. Taken together, these promising advantages of COS show the possibility of COS to be applied in renal diseases.

## 4. Materials and Methods

### 4.1. Chemical Reagents and Antibodies

Forskolin, STO-609, 3-(4,5-dimethylthiazol-2-yl)-2,5-diphenyltetrazolium bromide (MTT), dimethyl sulfoxide (DMSO), and DMEM/Ham F-12 were purchased from Sigma-Aldrich (St. Louis, MO, USA); 0.25% trypsin, fetal bovine serum (FBS), penicillin, and streptomycin were obtained from HyClone (Waltham, MA, USA); PureCol (3.1 mg/mL purified bovine collagen) was from Advanced BioMatrix (San Diego, CA, USA); bovine serum albumin (BSA) was from Calbiochem (San Diego, CA, USA). Indo-1 was from Life Technologies (Carlsbad, CA, USA). The rabbit primary antibodies against AMPK phosphorylated at Thri-172 (p-AMPK), AMPKα, and β-actin were purchased from Cell Signaling Technology (Boston, MA, USA). Goat anti-rabbit horseradish peroxidase (HRP)-conjugated secondary antibody was from Abcam (Cambridge, MA, USA).

### 4.2. Preparation of Chitosan Oligosaccharide

Chitosan oligosaccharide (COS), with an average molecular weight of ~5000 Da at degree of deacetylation (DD) of 90%, was prepared by enzymatic hydrolysis of chitosan derived from chitin, which was isolated from shrimp shells by chitinase enzymes, according to the Hackman method [53]. One hundred grams of *Penaeus vannamei* dried shells were soaked in 5 L of 1.5 N NaOH solution for 24 h, with three changes of the NaOH solution every 6 h with freshly prepared solution. The shells were then washed with 2 L of deionized water three times. After that, the shells were soaked in 5 L of 1.5 N HCl solution for 24 h, with three changes of the solution every 6 h. The shrimp chitin product was then washed with deionized water until neutral pH. The shrimp chitin was then soaked in 2 L of 50% (*w*/*w*) NaOH at room temperature for 5–7 days until over 90% deacetylation was achieved. The chitosan product was washed with deionized water until neutral pH, then air dried at 40–50 °C. The production of COS was achieved by the hydrolysis of 1% chitosan solution in 1% acetic acid, pH 4.5, with crude chitinase from *Bacillus licheniformis* SK-1 [54]. Thirty units of the enzyme per liter of the chitosan solution was used. Samples were taken out at different time points during a 3-h hydrolysis period. For the characterization, the sample with the appropriate average molecular weight of 5000 Da, as analyzed by gel permeation chromatography, was used in further experiments [55,56].

### 4.3. Cell Culture

Type I MDCK cells were the generous gift from Professor David N. Sheppard (University of Bristol, Bristol, UK). They were cultured in 1:1 Dulbecco’s modified Eagle’s medium/Ham’s F-12 nutrient mixture (DMEM/F-12), supplemented with 10% fetal bovine serum (FBS), 100 U/mL penicillin, 100 µg/mL streptomycin, 100 IU/mL insulin, 5 µg/mL transferrin, and 5 ng/mL selenium X in a humidified 95% O_2_/5% CO_2_ atmosphere at 37 °C.

### 4.4. MDCK Cyst Model

A 3D MDCK cyst model was established to imitate a renal cyst for the screening of bioactive compounds. In brief, MDCK cells at a density of 800 cells per well were grown in 24-well plates and suspended in 0.4 mL of 3 mg/mL collagen with the supplements including 10% of 10× minimum essential medium (MEM), 27 mM NaHCO_3_, 10 mM HEPES, 100 U/mL penicillin, and 100 µg/mL streptomycin, with the optimal pH of 7.4, adjusted by NaOH, in a CO_2_ incubator at 37 °C for 90 min. After gel setting, 1.5 mL of cultured media plus 10 µM of forskolin, an activator of adenylate cyclase (AC), which is used for stimulation of intracellular cAMP levels and in turn cyst formation, was added to each well. The photographs of the same individual MDCK cyst were taken for the measurement of the outer diameters of these cysts every other day (day 6, 8, 10, and 12). The cysts with ≥50 µm in diameter on day 6 were selected to be followed-up, and the media were changed every two days until day 12. At day 6, COS dissolved in 1% acetic acid at the concentrations of 10, 50, or 100 µg/mL were added into the medium in the continuous presence of forskolin. Then 1% acetic acid was used as the negative control, while 10 M CFTR_inh_-172 acted as a positive control. The cysts were observed at ×10 magnifications and the micrographs of individual cyst were obtained. They were considered to be “cysts” when their diameters were ≥50 µm, and the same cysts were followed with a mark on the bottom of the plate. Micrographs were taken by a Nikon TE 2000-S inverted microscope every two days from day 6 (before adding test compounds) to day 12. The diameters of cysts were measured using ImageJ software.

### 4.5. Cell Viability Assay

An MTT assay was performed to evaluate MDCK cell viability. In brief, MDCK cells were seeded in 96-well plates at a density of 10,000 cells per well and incubated in a humidified 95% O_2_/5% CO_2_ atmosphere at 37 °C. After reaching the cell confluence with ~80%, the cells were treated with 10, 50, or 100 µg/mL of COS dissolved in 1% acetic acid or vehicle control (1% acetic acid) for 24, 48, or 72 h. After the removal of the medium, serum-free MDCK medium containing water-soluble yellow dye MTT (5 mg/mL) was placed for 4 h in a humidified incubator with a 95% O_2_/5% CO_2_ atmosphere at 37 °C. In viable cells, the MTT was converted to a water-insoluble purple formazan by mitochondrial reductase enzyme. The formazan was solubilized in DMSO, and the absorbance was measured at optical density (OD) at 530 nm. MDCK cell viability was calculated as percentage of vehicle control.

### 4.6. Immunoblotting

Immunoblotting was performed to investigate the differential expression of the phosphorylated form of the proteins of interest. MDCK cells were plated onto 6-well plates at a density of 1 × 10^6^ cells per well. Confluent cells were treated with COS dissolved in 1% acetic acid at the concentrations of 10, 50, or 100 µg/mL or 1% acetic acid (vehicle control) for a dose–response study. Proteins were harvested using a radioimmunoprecipitation (RIPA) lysis buffer containing 1% Triton X-100, 50 mM Tris-HCl (pH 7.4), 150 mM NaCl, 1 mM EDTA, 1 mM NaF, 1 mM Na_3_VO_4_, 1 mM phenylmethylsulfonyl fluoride (PMSF), and protease inhibitor (PI) cocktail. Then, sodium dodecyl sulfate polyacrylamide gel electrophoresis (SDS–PAGE) was used for separating the proteins. After that, those proteins were transferred to a nitrocellulose membrane before blocking non-specific binding proteins by 5% BSA for 1 h at room temperature. The membranes were incubated with antibodies against proteins of interest at 4 °C overnight and washed with tris-buffered saline with 0.1% Tween 20 detergent (TBST) solution at least four times. After that, the membrane was incubated with horseradish peroxidase (HRP)-conjugated anti-rabbit IgG antibodies. The immunoblot was visualized using a chemiluminescence detection method. Band intensity of immunoblots of the protein of interest was normalized with that of β-actin.

### 4.7. Intracellular Calcium Measurement

A fluorometric-based assay was executed for measuring the intracellular Ca^2+^ concentrations ([Ca^2+^]_i_) in MDCK cells using indo-1 as a calcium indicator. MDCK cells were grown in 6-well plates. at a density of 1 × 10^6^ cells per well. Confluent MDCK cells were trypsinized, resuspended, and washed with phosphate buffer saline (PBS) solution three times. After that, the cells were incubated with 1 mM of indo-1 for 1 h in the dark at 37 °C and washed at least three times with fresh calcium buffer composed of 1 mM CaCl_2_, 137.93 mM NaCl, 0.338 mM Na_2_HPO_4_, 4.17 mM NaHCO_3_, 5.33 mM KCl, 0.441 mM KH_2_PO_4_, 5.56 mM D-glucose, and 1% (*w*/*v*) BSA. The ratio of fluorescence emission at 405 nm (Ca^2+^-bound indo-1) and at 490 nm (Ca^2+^-free indo-1), with fluorescence excitation at 338 nm, was monitored by a FP-6200 spectrofluorometer (JASCO, Essex, UK). The magnitude in the indo-1 fluorescence ratio reflected the increment in the level of [Ca^2+^]_i_.

### 4.8. Statistical Analysis

All experimental data were expressed as the mean ± standard error (S.E.M.). Statistical analysis for determining the difference between the control and experimental groups was performed by one-way analysis of variance (ANOVA) test, followed by Bonferroni’s method for multiple comparisons, using GraphPad Prism software (GraphPad Software, San Diego, CA, USA). The *p*-value of <0.05 was considered statistically significant.

## 5. Conclusions

To summarize, our present study delineates the novel potential of COS, with an average molecular weight of ~5000 Da at a degree of deacetylation (DD) of 90%, in retarding renal epithelial cyst progression. The underlying mechanism of COS in inhibiting renal cyst growth involves activation of the Ca^2+^-CaMKKβ-AMPK pathways. Further investigations for the effect of COS on reducing renal cyst growth in animal models of PKD are required to warrant potential utility of COS in PKD treatment.
